# SGK1 Mediates Hypoxic Pulmonary Hypertension through Promoting Macrophage Infiltration and Activation

**DOI:** 10.1155/2019/3013765

**Published:** 2019-11-13

**Authors:** Xin Xi, Jing Zhang, Jian Wang, Yuqin Chen, Wenmei Zhang, Xiaoping Zhang, Jie Du, Guangfa Zhu

**Affiliations:** ^1^Department of Pulmonary and Critical Care, Beijing Anzhen Hospital, Capital Medical University, Beijing, China; ^2^Beijing Institute of Heart, Lung and Blood Vessel Diseases, Key Laboratory of Remodeling-Related Cardiovascular Diseases, Ministry of Education, Beijing Collaborative Innovation Center for Cardiovascular Disorders, Beijing, China; ^3^State Key Laboratory of Respiratory Diseases, Guangzhou Institute of Respiratory Disease, The 1st Affiliated Hospital of Guangzhou Medical University, Guangzhou, China

## Abstract

Inflammation plays a pivotal role in the development of pulmonary arterial hypertension (PAH). Meanwhile, serum glucocorticoid-regulated kinase-1 (SGK1) has been considered to be an important factor in the regulation of inflammation in some vascular disease. However, the role of SGK1 in hypoxia-induced inflammation and PAH is still unknown. WT and SGK1^−/−^ mice were exposed to chronic hypoxia to induce PAH. The quantitative PCR and immunohistochemistry were used to determine the expression of SGK1. The right ventricular hypertrophy index (RVHI), RV/BW ratio, right ventricle systolic pressure (RVSP), and percentage of muscularised vessels and medical wall thickness were measured to evaluate PAH development. The infiltration of macrophages and localization of SGK1 on cells were examined by histological analysis. The effects of SGK1 on macrophage function and cytokine expression were assessed by comparing WT and SGK1^−/−^ macrophages in vitro. SGK1 has high expression in hypoxia-induced PAH. Deficiency of SGK1 prevented the development of hypoxia-induced PAH and inhibited macrophage infiltration in the lung. In addition, SGK1 knockout inhibited the expression of proinflammatory cytokines in macrophages. SGK1-induced macrophage activation and proinflammatory response contributes to the development of PAH in hypoxia-treated mice. Thus, SGK1 might be considered a promising target for PAH treatment.

## 1. Introduction

Pulmonary arterial hypertension (PAH) is a progressive and life-threatening disease with a poor prognosis [1]. PAH is characterized by pulmonary vasoconstriction and increased pulmonary vascular resistance leading to right ventricular failure, fluid overload, and death [2]. The major histopathological feature of PAH is vascular wall remodeling, and the remodeling process includes intima proliferation, the medial and adventitial layer hypertrophy, and extracellular matrix deposition [3]. Pulmonary arteries exhibit complex structural and functional changes in PAH, and various cell types and growth factors are involved in the development of PAH [4]. Although PAH is primarily considered to be a vascular disease, there is a well-established link between PAH and inflammation [5], and evidence from many clinical and basic researches suggests that inflammation plays an important role in the process of PAH [6].

Pulmonary vascular lesions in patients with PAH and the animal models of pulmonary hypertension are characterized by infiltration of many inflammatory cells, including T lymphocytes, B lymphocytes, macrophage, dendritic cells, and mast cells, around the blood vessels [6]. Among these inflammatory cells in PAH, the monocytes/macrophages are more often associated with the disease [7, 8]. CD68^+^ macrophages are observed in advanced obliterative plexiform lesions in both experimental and clinical PAH [8–10]. Depletion or inactivation of macrophages may inhibit PAH in a variety of model systems, including experimentally induced hypoxic PAH and portopulmonary hypertension [11, 12]. The activation of macrophages promotes vascular injury and the development of angioobliterative pulmonary hypertension by inducing pulmonary artery endothelial cell injury and apoptosis as well as smooth muscle cell proliferation [12]. It is still not clear which key factors regulate macrophage activation and ultimately promote PAH development.

Serum glucocorticoid-regulated kinase 1 (SGK1) belongs to the cAMP-dependent protein kinase 1/cGMP-dependent protein kinase/protein kinase C family [13]. The SGK1 promoter contains a number of transcription factor binding sites that are responsible for the stimulated regulation of SGK1 expression [14]. Because of these binding sites, SGK1 is primarily transactivated in response to various hormonal and nonhormonal extracellular stimuli [15]. Under physiological conditions, the majority of cells express low levels of SGK1, and the expression of SGK1 in some cells is much higher under certain pathophysiological conditions. Tissue ischemia, tissue hypoxia, and tumor necrosis factor-*α* (TNF-*α*) and interleukin-6 (IL-6) and other inflammatory stimuli induce high SGK1 expression [14, 16–18]. SGK1 participates in various biological activities such as cell proliferation, cell apoptosis, and ion channel regulation, and it plays an important role in the regulation of inflammation [14, 19–21]. SGK1 is considered to be an important factor regulating inflammation and vascular disease development [22–24]. However, the role of SGK1 in hypoxia-induced PAH remains unclear.

In this study, we investigated the role of SGK1 in response to chronic hypoxia. Our results indicate that deficiency of SGK1 attenuates hypoxia-induced pulmonary hypertension. This attenuation was associated with inhibition of vascular remodeling and reduction in the inflammatory response. Additionally, we show that SGK1 deficiency inhibits the activation of macrophages in vitro. These data provide first proof to describe an important role of SGK1 in macrophage activation and hypoxia-induced PAH development.

## 2. Materials and Methods

### 2.1. Mouse Models

SGK1 knockout (SGK1^−/−^) and littermate control (WT) mice were generated as before [25]. At the age of 10–12 weeks, male SGK1^−/−^ mice and their WT littermates had free access to food and were housed under specific pathogen-free conditions with a 12 : 12 hour light–dark cycle between 20 and 24°C. WT and SGK1^−/−^ mice were maintained with standard laboratory chow and water ad libitum. All animal housing and experimental protocols were approved by the Animal Care and Use Committee of Capital Medical University and conformed to the US National Institutes of Health Guide for the Care and Use of Laboratory Animals (NIH Publication No. 8023, revised 1978).

### 2.2. Exposure to Chronic Hypoxia

WT and SGK1^−^/^−^ mice were randomly assigned to the normoxia group (N) and hypoxia group (H). The normoxia group was exposed to room air, whereas the hypoxia-group mice were placed in a ventilated chamber with 10% O_2_ and CO_2_ < 0.5% environment. The environment was established by a mixture of room air and nitrogen with a detector (RCI Hudson, Anaheim, CA, USA) which monitored and controlled the fractional concentration of O_2_ automatically. The chamber was opened every 3 to 4 days for changing cage and replenishing food and water.

### 2.3. Hemodynamics and Ventricular Weight Measurements

After 4 weeks of normoxia or hypoxic exposure, right ventricular systolic pressure (RVSP) and right ventricular hypertrophy index (RVHI) was measured as previously described [26]. Briefly, right ventricular pressure (RVP) was measured with a pressure transducer catheter and AcqKnowledge software (Biopac Systems Inc., CA, USA) by a 23-gauge needle via the diaphragm into the right ventricle (RV). The RVSP was then recorded as a surrogate for pulmonary artery pressure. Mouse hearts were excised and right ventricular hypertrophy was evaluated by right ventricular hypertrophy index (RVHI), which was the wet weight ratio of the right ventricle (RV) to left ventricle (LV) plus septum (S). The RV/BW ratio was obtained through measuring the right ventricular mass and body weight.

### 2.4. Histological Analysis

After anesthesia, the mice were perfused with PBS through LV. Then, the lung tissues of mice were harvested, fixed in formalin, and embedded in paraffin. Lung cross sections at 5 *μ*m intervals were stained by hematoxylin and eosin (H&E) with standard procedures. For immunohistochemistry, lung sections were incubated with antibodies against SGK1 (1 : 100, Abcam, Cambridge, MA), *α*-smooth muscle actin (*α*-SMA, 1 : 100, ZSGB-BIO, Beijing, China), or Mac3 (1 : 200, Santa Cruz Biotechnology, Dallas, TX) at 4°C overnight, then, with secondary antibodies at 4°C for 1 hour and detected with 3,3-diaminobenzidine, and sections were counterstained with hematoxylin.

Morphometric analysis to quantify pulmonary arterial (PA) remodeling was made on the distal small PA by measuring PA wall thickness or the thickness of the smooth muscle layer in H&E and *α*-SMA stain images of lung cross sections captured by a Nikon Eclipse Ni microscope (Nikon, Tokyo, Japan) and analyzed by NIS-Elements AR 4.0 software (Nikon). Muscularised vessel % was calculated as the number of muscularised vessels/total number of vessels counted per section × 100 and medial wall thickness % (MWT%) (the ratio of the average medial thickness divided by the average vessel radius) was assessed in 30 muscular PA with an external diameter of 50–100 *μ*m per lung section. Mac-3-positive cells per vessel were determined by counting 30 to 35 vessels per mouse. Mac-3-positive cells per 30 HPF were counted in 30 high-power fields (magnification: 400x) per mouse.

For immunofluorescence, lung sections or cells were labeled with primary antibodies F4/80 (1 : 100, Abcam) and SGK1 (1 : 100, Abcam) and then incubated with Alexa Fluor 555- and 488- conjugated secondary antibody (1 : 500, Thermo Fisher). Images were captured by a Leica TSC-SP5 laser-scanning confocal microscope (Leica, Wetzlar, Germany).

### 2.5. Macrophage Culture

Bone marrow-derived macrophages were prepared with a modification as described previously [27]. In brief, bone marrow cells were obtained by flushing from the dissected femurs and tibias, and cells were resuspended and cultured in RPMI 1640 medium (Life Technologies, Grand Island, NY) supplemented with 10% FBS in the presence of 50 ng/ml recombinant murine M-CSF. Macrophages were stimulated with 50 ng/ml LPS (Sigma-Aldrich, St. Louis, MO) for 4 h.

### 2.6. Quantitative RT-PCR Analysis

Bone marrow-derived macrophage RNA was extracted by TRIzol reagent according to the manufacturer's protocol (Thermo Fisher). Equal amounts of RNA (1 *μ*g) were added to reverse transcriptase reaction mix with oligo-dT primers (Promega, Southampton, UK). SYBR Premix Ex Taq (TaKaRa, Shiga, Japan) was used to perform quantitative real-time PCRs with IQ5 Multicolor Real-Time PCR Detection System (Bio-Rad, Hercules, CA). The following primers were used: SGK1 (5′-CGTTCGGTCTTCATTCAC-3′ forward; 5′-TTATAGATCCAGGGTGGC-3′ reverse), MCP-1 (5′-CTGAAGCCAGCTCTCTCTTCCT-3′ forward; 5′-CAGGCCCAGAAGCATGACA-3′ reverse), TNF-*α* (5′-CACAAGATGCTGGGACAG-TGA-3′ forward; 5′-TCCTTGATGGTGGTGCATGA-3′ reverse), IL-1*β* (5′-CCATGG-CACATTCTGTTCAAA-3′ forward; 5′-GCCCATCAGAGGCAAGGA-3′ reverse), IL-10 (5′-CCAGGGAGATCCTTTGATGA-3′ forward; 5′-CATT-CCCAGAGGAATTGCAT-3′ reverse), and GAPDH (5′-CATGGCCTTCCGTGTTCCTA-3′ forward; 5′-GCGGCACGTCAGATCCA-3′ reverse).

### 2.7. Cytometric Bead Array (CBA)

To measure the release of cytokines (IFN-*γ*, IL-6, IL-12, MCP-1, TNF, and IL-10), we used the BD CBA mouse inflammation kit (BD Biosciences, San Jose, CA). The macrophage culture supernatant was collected 4 hours after LPS stimuli, and the assays were performed according to the manufacturer's instruction. The results were analyzed with FCAP Array Version 3.0 (Soft Flow, Duesseldorf, Germany).

### 2.8. Statistical Analysis

All data are expressed as mean ± SD. Statistics were calculated with SPSS computer software for Windows (version 13.0; SPSS, Chicago, IL). The Student *t*-test was used to compare data between two groups in each experiment. One-way ANOVA, followed by a Bonferroni–Dunn test, was used to compare data between three or more groups in each experiment. Results were considered to represent significant differences at *p* < 0.05.

## 3. Results

### 3.1. Hypoxia-Induced PAH Increases SGK1 Expression in Mouse Lung

**Figure 1:**
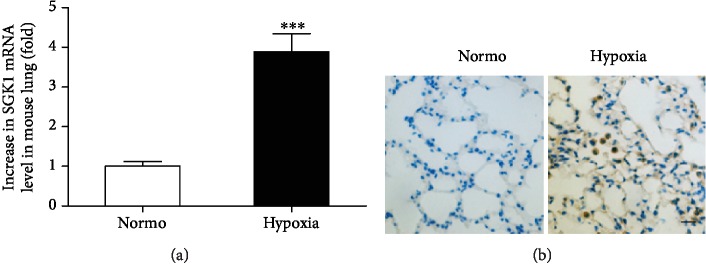
Hypoxia-induced PAH increased expression of SGK1 in mice. (a) Lung mRNA levels for SGK1 were measured using quantitative real-time PCR from mice exposed to 3 days of normoxia or hypoxia. GAPDH was used to normalize the quantitative real-time data. Results are expressed as relative fold changes compared with the normoxia group (*n* = 6 − 7 in each group). (b) Immunohistochemical analysis of SGK1 expression in lung sections from mice exposed to 7 days of normoxia or hypoxia. Scale bar = 10 *μ*m. ^∗∗∗^*p* < 0.001.

To investigate the role of SGK1 in the development of chronic PAH, we examined SGK1 expression in the lungs of mouse with hypoxia. The mRNA level of SGK1 was upregulated in the lungs of hypoxia-induced mice compared to the lungs of normoxia-induced mice (Figure 1(a)). Immunohistochemistry revealed that SGK1-positive cells had infiltrated into the alveolar space of the lungs in hypoxia-induced mice (Figure 1(b)). Thus, SGK1 expression and activation may play an important role in the development of hypoxia-induced PAH.

### 3.2. SGK1 Deficiency Inhibits Hypoxia-Induced PAH Development

**Figure 2:**
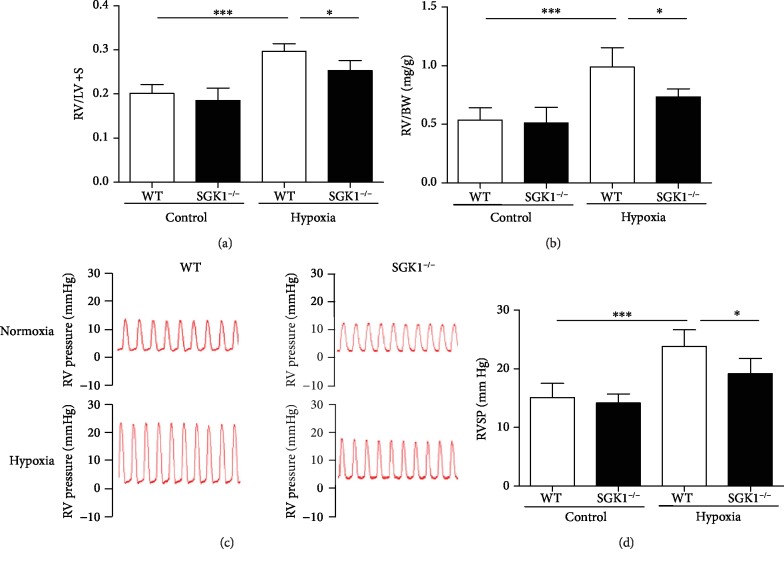
SGK1 deficiency alleviated the development of PAH in mice. (a) Graphs of right ventricular hypertrophy index (right ventricle/left ventricle plus septum weight, RV/(LV+S)) from mice exposed to 28 days of normoxia or hypoxia. (b) Graphs of right ventricular mass to body weight ratio (RV/BW) from mice exposed to 28 days of normoxia or hypoxia. (c) Representative images of right ventricular systolic pressure (RVSP) waves (red) from WT and SGK1^−/−^ mice exposed to 28 days of normoxia or hypoxia. (d) Graphs of right ventricular systolic pressure (RVSP). *n* = 6 in each group, ^∗^*p* < 0.05, ^∗∗∗^*p* < 0.001.

Given that SGK1 was upregulated in the lungs of hypoxia-induced mice, we sought to determine the effect of SGK1 on the development of PAH. Both WT- and SGK1-deficient (SGK1^−/−^) mice were exposed to hypoxia or normoxia for 4 weeks. Hypoxia treatment markedly increased RV/(LV+S) and RV/BW ratios in both WT and SGK1^−/−^ mice compared to normoxia-treated mice, while SGK1^−/−^ mice significantly reduced RV/(LV+S) and RV/BW ratios compared to WT mice under hypoxic conditions (Figures 2(a) and 2(b)). RVSP was much higher in hypoxic WT and SGK1^−/−^ mice than in normoxic WT and SGK^−/−^ mice. However, this increase in RVSP was inhibited in SGK1^−/−^ mice compared to WT mice under hypoxic conditions (Figures 2(c) and 2(d)). These results suggest that SGK1 aggravates right ventricular systolic pressure and right ventricular hypertrophy in hypoxia-induced PAH.

### 3.3. SGK1 Deficiency Reduces Pulmonary Arterial Remodeling in Hypoxia-Induced PAH

**Figure 3:**
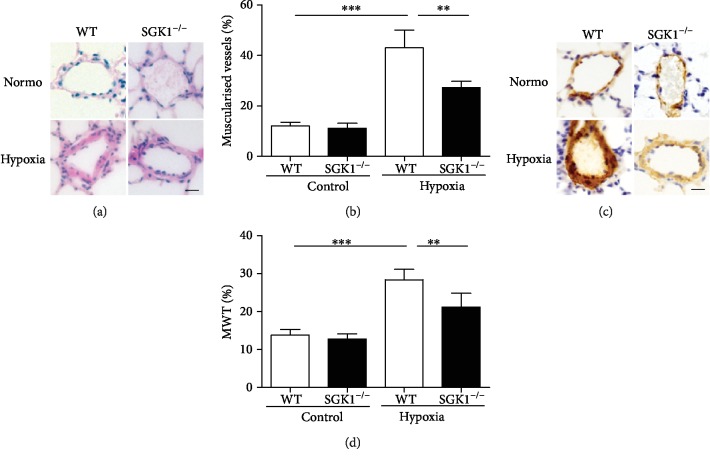
SGK1 deficiency alleviated pulmonary arterial remodeling of PAH in mice. (a) Representative H&E images of pulmonary arteries from mice exposed to 28 days of normoxia or hypoxia. (b) Graphs of muscularised vessels calculated as the number of muscularised vessels to total number of vessels. (c) Representative immunohistochemical staining of *α*-SMA images in pulmonary arteries from mice exposed to 28 days of normoxia or hypoxia. (d) Graphs of medial wall thickness calculated as the average medial thickness to the average vessel radius. Scale bar = 10 *μ*m. *n* = 6 in each group, ^∗∗^*p* < 0.01, ^∗∗∗^*p* < 0.001.

To determine the effects of SGK1 on pulmonary arterial remodeling, we investigated the percentage of muscularised vessels (the number of muscularised vessels/total number of vessels) by H&E staining and the percentage of medial wall thickness (MWT%, the ratio of the average medial thickness divided by the average vessel radius) by immunohistochemical staining of *α*-SMA. Under hypoxia, the muscularised vessels were increased in WT and SGK1^−/−^ mice, while SGK1^−/−^ mice significantly decreased muscularised vessels compared to WT mice (Figures 3(a) and 3(b)). The pulmonary artery wall area to total area ratio was significantly higher in the PAH model groups compared with the control groups, while SGK1 deficiency significantly reduced the ratio of the wall area to total area (Figures 3(c) and 3(d)). Therefore, SGK1 promotes pulmonary arterial remodeling in hypoxia-induced PAH.

### 3.4. SGK1 Deficiency Inhibits Inflammatory Response in Hypoxia-Induced PAH

**Figure 4:**
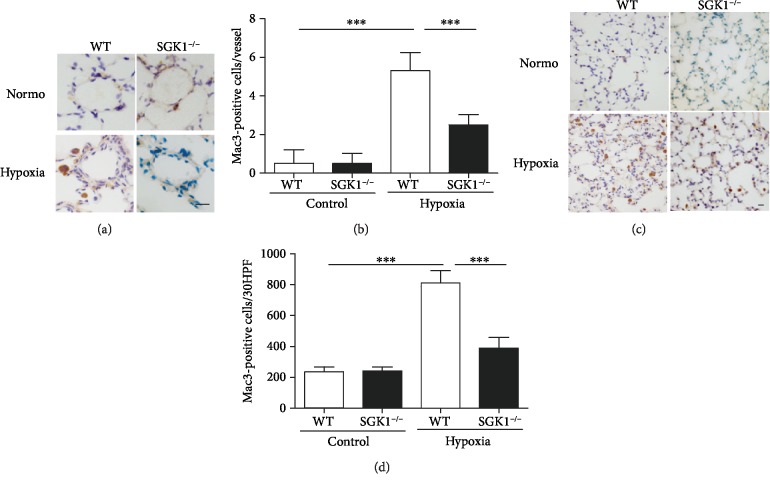
SGK1 deficiency decreased macrophage accumulation in hypoxic mice. (a) Representative immunohistochemical staining of Mac3 images in pulmonary arteries from mice exposed to 28 days of normoxia or hypoxia. (b) Graphs of Mac3-positive cells per vessel. (c) Representative immunohistochemical staining of Mac3 images in the lungs from mice exposed to 28 days of normoxia or hypoxia. (d) Graphs of Mac3-positive cells per 30 HPF. Scale bar = 10 *μ*m. *n* = 6 in each group, ^∗∗∗^*p* < 0.001.

Previous studies have shown that inflammation exacerbates pulmonary vascular remodeling during PAH [28] and depletion or inactivation of macrophages can inhibit PAH development [11, 12]. For these reasons, the effect of SGK1 on regulating pulmonary inflammation was investigated by detecting macrophage infiltration. Macrophage infiltration was detected by immunohistochemical staining of Mac3 (macrophage marker), and macrophage accumulation was quantitated by counting the number of macrophages around the blood vessels and the overall number of macrophages in the lung. Under hypoxia, the number of macrophages around the blood vessels was increased in WT and SGK1^−/−^ mice, while SGK1^−/−^ mice significantly decreased the number of macrophages around the blood vessels compared to WT mice (Figures 4(a) and 4(b)). SGK1 deficiency also significantly inhibited macrophage infiltration in the lung under hypoxic conditions (Figures 4(c) and 4(d)). Thus, SGK1 promotes macrophage infiltration in hypoxia-induced PAH.

### 3.5. SGK1 Expression on Macrophage Is Essential for PAH Development

**Figure 5:**
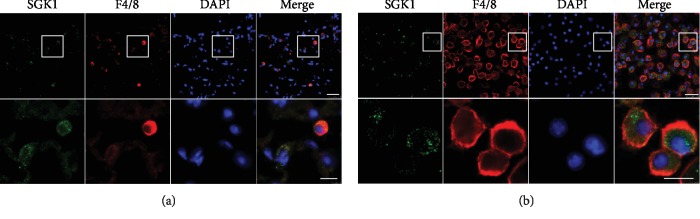
SGK1 expressed on macrophages in hypoxia-induced lungs and macrophages derived from the bone marrow. (a) Representative of lung tissue from WT mice exposed to 7 days of hypoxia. Top panels present the low-magnification images of SGK1 (green), F4/80 (red), and DAPI (blue). Scale bar = 25 *μ*m. Bottom panels present the high-magnification insets from the white boxes of the top panels. Scale bar = 10 *μ*m. (b) Representative of bone marrow-derived macrophage. Top panels present the low-magnification images of SGK1 (green), F4/80 (red), and DAPI (blue). Scale bar = 25 *μ*m. Bottom panels present the high-magnification insets from the white boxes of the top panels. Scale bar = 10 *μ*m.

We detected the localization of SGK1 in hypoxia-induced lungs by double immunofluorescence staining. The results showed that SGK1 mainly colocalize with F4/80, a marker of macrophages (Figure 5(a)). We isolated macrophages from bone marrow and detected SGK1 expression by immunofluorescence staining and found SGK1 and F4/80 were colocalized in bone marrow-derived macrophages (Figure 5(b)). Thus, SGK1 is mainly expressed on macrophages in hypoxia-induced lungs and macrophages isolating from the bone marrow.

### 3.6. SGK1 Promotes Macrophage Activation and Inflammatory Response In Vitro

**Figure 6:**
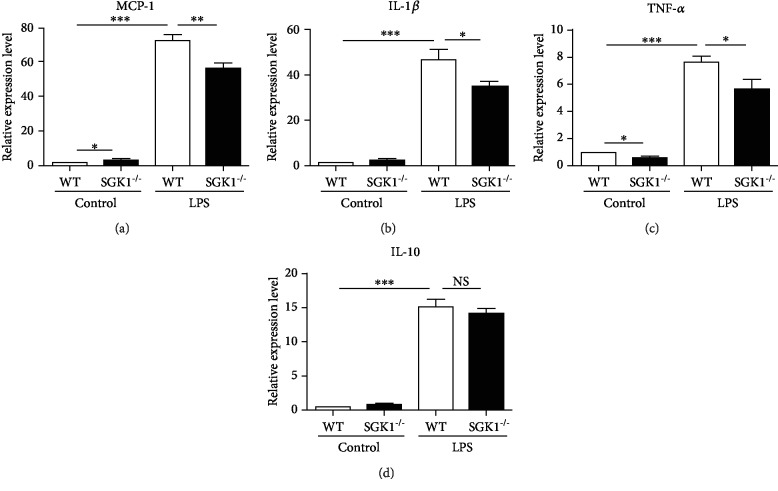
SGK1 deficiency decreased proinflammatory cytokine expression in vitro. Expression of MCP-1 (a), IL-1*β* (b), TNF-*α* (c), and IL-10 (d) was analyzed by quantitative PCR in bone marrow-derived macrophage stimulated with 50 ng/ml LPS for 4 hours. GAPDH was used to normalize the quantitative real-time data. Results are expressed as relative fold changes compared with the control group. Data represent at least three independent experiments. ^∗^*p* < 0.05, ^∗∗^*p* < 0.01, ^∗∗∗^*p* < 0.001.

**Figure 7:**
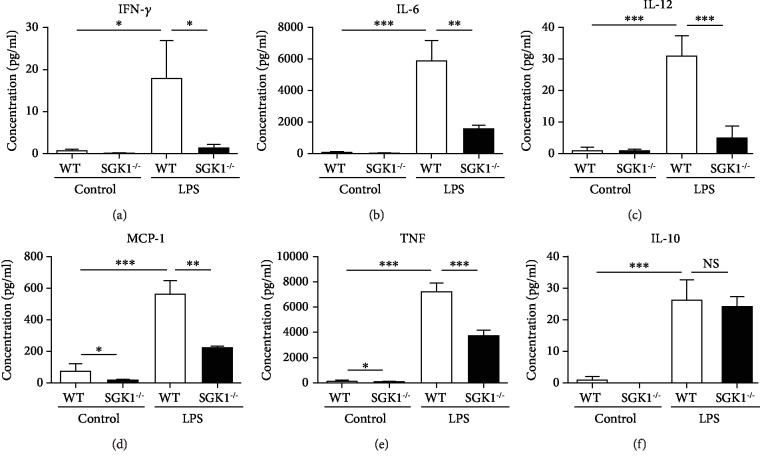
SGK1 deficiency decreased proinflammatory cytokine secretion in vitro. Secretion of IFN-*γ* (a), IL-6 (b), IL-12 (c), MCP-1 (d), TNF (e), and IL-10 (f) was analyzed by cytometric bead array (CBA) in bone marrow-derived macrophage stimulated with 50 ng/ml LPS for 4 hours. Data represent at least three independent experiments. ^∗^*p* < 0.05, ^∗∗^*p* < 0.01, ^∗∗∗^*p* < 0.001.

We next explored the mechanisms of inhibiting pulmonary inflammatory response in SGK1^−/−^ mice with PAH. As SGK1 was mainly expressed on macrophages and macrophages were involved in the process of inflammation and tissue injury by secreting numerous biologically active molecules, we detected the secretion of cytokines in SGK1^−/−^ macrophages with LPS stimulation. Cytokine expressions of WT and SGK1^−/−^ macrophages derived from bone marrow with LPS stimulation were analyzed by quantitative PCR. As shown in Figure 6, LPS significantly increased the expression of MCP-1, IL-1*β*, TNF-*α*, and IL-10 from cultured WT macrophages, and SGK1 knockout reduced the expression of proinflammatory cytokines, MCP-1, IL-1*β*, and TNF-*α*. Meanwhile, SGK1 knockout had no significant effect on the expression of anti-inflammatory cytokine, IL-10 (Figure 6(d)). The concentrations of cytokines in the supernatant of WT and SGK1^−/−^ macrophages with LPS stimulation were analyzed by BD cytometric bead array (CBA) with a mouse inflammation kit. Consistent with the results of mRNA expression, SGK1 knockout inhibited the production of proinflammatory cytokines, IFN-*γ*, IL-6, IL-12, MCP-1, and TNF, and had no significant effect on the production of anti-inflammatory cytokine, IL-10 (Figure 7). Therefore, SGK1 promotes macrophage activation and proinflammatory response in vitro.

## 4. Discussion

We provide the first evidence for the critical role of SGK1 in hypoxia-induced PAH by regulating proinflammation response. Hypoxia-induced PAH significantly increased the expression of SGK1 in mouse lung. Lack of SGK1 markedly ameliorated hypoxia-induced PAH development and pulmonary arterial remodeling. SGK1 deficiency also inhibited inflammatory response in PAH. Moreover, SGK1 is expressed on macrophages in hypoxia-induced lungs in vivo, and SGK1 deletion in bone marrow-derived macrophages inhibited secretion of proinflammatory cytokines in vitro. Our findings suggest that SGK1 plays an important role in PAH development and may be a promising therapeutic target for PAH therapy.

Previous studies have shown that perivascular immunity and inflammation play a key role in the pathogenesis of idiopathic PAH [29]. Infiltrates of inflammatory cells, including macrophages, dendritic cells, and T and B cells, have been detected in pulmonary vascular lesions of patients with severe pulmonary hypertension [28]. And leukocytes and inflammatory mediators have been detected in experimental PAH models such as monocrotaline-treated rats [9, 30] and hypoxia-induced mice [8, 31]. In addition to infiltration of pulmonary immune cells, elevated levels of serum inflammatory cytokines and chemokines have been reported in patients with PAH [32], and some elevated levels of inflammatory cytokines, such as IL-1*β* and IL-6, have been shown to predict survival in idiopathic and familial pulmonary arterial hypertension [33]. Previous studies have demonstrated that inflammation may contribute to the development of PAH, and anti-inflammatory treatment may be a promising strategy for severe PAH [34]. Activated CD68^+^ macrophages accumulated in the pulmonary arteries of hepatopulmonary syndrome and macrophage depletion by intravenous injections of either gadolinium chloride or liposomal clodronate prevented and reversed hepatopulmonary syndrome [11]. In a rat model of severe pulmonary hypertension, macrophages accumulated around pulmonary arterioles, and depleting CD68^+^ macrophages prevented the development of pulmonary hypertension [28]. Thus, depletion or inactivation of macrophages may have a therapeutic potential in pulmonary hypertension. In our study, hypoxia stimulation induced macrophage infiltration in the lung of WT mice, and deficiency of SGK1 inhibited macrophage infiltration. Therefore, SGK1 deletion inhibited the development of PAH and was likely related to suppressed macrophage infiltration. Moreover, deficiency of SGK1 suppressed the activation of macrophages by inhibiting the macrophage expression of proinflammatory cytokines. Our results uncover a possible role for SGK1 in PAH pathogenesis and identify a key molecular that may be amenable to therapeutic targeting.

SGK1, a downstream effector of the phosphoinositide-3 kinase cascade, is a serine-threonine kinase and can be activated by various stimuli, such as steroids and peptide hormones like mineralocorticoids, growth factors like TGF-*β*, and cytokines like IL-6 [16, 35]. The expression of SGK1 is highly variable and subject to regulation by a wide variety of triggers. SGK1 stimulates various renal tubular ion channels and transporters and is therefore involved in the regulation of renal electrolyte excretion [35]. SGK1 plays an important role in inflammatory responses in cardiac fibroblasts triggered by mechanical stretch [36]. SGK1 promotes survival, invasiveness, motility, epithelial to mesenchymal transition, and adhesiveness of tumor cells [37]. SGK1 contributes to monocyte/macrophage migration and MMP-9 transcription during atherogenesis [23]. In our study, we have found SGK1 expressed on macrophage after pulmonary arterial hypertension (Figure 5(a)), and lack of SGK1 markedly inhibited inflammatory response and ameliorated hypoxia-induced PAH. Our findings suggest that SGK1 expressed on macrophages plays an important role in PAH development.

Previous studies have demonstrated that SGK1 as an important factor for regulating inflammation and vascular disease development [22–24]. In cardiac ischemia-reperfusion injury, SGK1 promotes the release of proinflammatory factors and reduces the level of anti-inflammatory factors [22]. In other vascular diseases, SGK1 also have effects on the inflammation of the disease by regulating macrophage activity [23, 24]. Despite the wide tissue distribution of SGK1 and its sensitivity to various stimuli, its role in hypoxia-induced pulmonary hypertension was not fully defined. In our study, we found SGK1 induced in hypoxia-induced pulmonary hypertension and SGK1 deletion in mice inhibited the development of pulmonary hypertension. Previous studies have demonstrated that increased expression of SGK1 is associated with inflammation. SGK1 played a key role in the induction of pathogenic Th17 cells and facilitated tissue inflammation [20]. SGK1 inhibition reduced proinflammatory cytokine IL-17 and reduced anti-inflammatory cytokines IL-10 and IL-27 in the ischemic-reperfused heart [22]. In a transgenic zebrafish model, SGK1 gene disruption delayed inflammation resolution, without interrupting neutrophil infiltration in vivo [21]. These results are consistent with our results in the model of hypoxia-induced pulmonary inflammation, where we showed that the macrophage infiltration was significantly decreased in pulmonary tissue of SGK1 knockout mice compared to that of WT mice. And our findings also suggest an important role of SGK1 in the regulation of pulmonary inflammation and PAH development.

SGK1 can phosphorylate the cAMP-responsive element-binding protein (CREB) and interfere with CREB-dependent gene transcription [38]. SGK1 also could enhance the activity of NF*κ*B. On the one hand, SGK1 activates the kinase I*κ*B kinase beta (IKK*β*), which subsequently phosphorylates and degrades I*κ*B, an inhibitor of NF*κ*B, and promotes NF*κ*B entry into the nucleus and turns on gene transcription [14, 39]. On the other hand, SGK1 phosphorylates and activates NDRG1, which is specific target of SGK1. NDRG1 could downregulate NF*κ*B signaling [40]. For NF*κ*B regulating the expression of various cytokines, SGK1 could regulate cytokine expression by activating NF*κ*B. Thus, in our study, SGK1 may promote cytokine expression by activating NF*κ*B.

Macrophages respond to environmental signals with plasticity and undergo different forms of polarized activation, which can be broadly divided into classically activated (M1) macrophages and alternatively activated (M2) macrophages. IFN-*γ* generated by Th1 cells activates classical macrophages expressing proinflammatory cytokines TNF-*α*, IL-1*β*, and IL-6. Th2 cytokines, IL-4, and/or IL-13 activate alternative macrophages expressing IL-10 [41]. In our study, we found that SGK1 deletion inhibited the expression of proinflammatory cytokines, such as IL-6, TNF-*α*, and IL-1*β*. Circulating levels of these cytokines, IL-6, TNF-*α*, and IL-1*β*, were abnormally elevated in patients with PAH, and all of these cytokines were associated with the poor clinical outcomes in PAH patients [33, 42]. Moreover, both IL-6-overexpressing mice and TNF-*α*-overexpressing mice induced spontaneously pulmonary hypertension and pulmonary vascular remodeling [43, 44], while IL-6 knockout inhibited the development of pulmonary hypertension induced by chronic hypoxia [45]. Inhibition of the IL-1 signaling pathway by treatment of IL-1 receptor antagonist reduced pulmonary hypertension in rat [46]. These previous studies suggest that IL-6, TNF-*α*, and IL-1*β* may be actively involved in pulmonary hypertension. Thus, in our results, the inhibitory effect of SGK1 knockdown on PAH is likely through suppressing the expression of crucial cytokines, such as IL-6, TNF-*α*, and IL-1*β*.

## 5. Conclusion

In summary, we provide the evidences that SGK1 promotes the development of PAH induced by chronic hypoxia. SGK1 also plays an important role in the inflammation of hypoxia-induced PAH, and deletion of SGK1 is found to attenuate inflammation-associated PAH. Our findings highlight a potential option for therapeutic intervention of PAH.

## Data Availability

The data used to support the findings of this study are included within the article.

## References

[1] Park Y. M., Chung W. J., Choi D. Y. (2014). Functional class and targeted therapy are related to the survival in patients with pulmonary arterial hypertension. *Yonsei Medical Journal*.

[2] Lai Y. C., Potoka K. C., Champion H. C., Mora A. L., Gladwin M. T. (2014). Pulmonary arterial hypertension: the clinical syndrome. *Circulation Research*.

[3] Stenmark K. R., Fagan K. A., Frid M. G. (2006). Hypoxia-induced pulmonary vascular remodeling: cellular and molecular mechanisms. *Circulation Research*.

[4] Crosswhite P., Sun Z. (2014). Molecular mechanisms of pulmonary arterial remodeling. *Molecular Medicine*.

[5] Hassoun P. M., Mouthon L., Barbera J. A. (2009). Inflammation, growth factors, and pulmonary vascular remodeling. *Journal of the American College of Cardiology*.

[6] Rabinovitch M., Guignabert C., Humbert M., Nicolls M. R. (2014). Inflammation and immunity in the pathogenesis of pulmonary arterial hypertension. *Circulation Research*.

[7] Vergadi E., Chang M. S., Lee C. (2011). Early macrophage recruitment and alternative activation are critical for the later development of hypoxia-induced pulmonary hypertension. *Circulation*.

[8] Frid M. G., Brunetti J. A., Burke D. L. (2006). Hypoxia-induced pulmonary vascular remodeling requires recruitment of circulating mesenchymal precursors of a monocyte/macrophage lineage. *The American Journal of Pathology*.

[9] Dorfmuller P., Perros F., Balabanian K., Humbert M. (2003). Inflammation in pulmonary arterial hypertension. *The European Respiratory Journal*.

[10] Tuder R. M., Groves B., Badesch D. B., Voelkel N. F. (1994). Exuberant endothelial cell growth and elements of inflammation are present in plexiform lesions of pulmonary hypertension. *The American Journal of Pathology*.

[11] Thenappan T., Goel A., Marsboom G. (2011). A central role for CD68(+) macrophages in hepatopulmonary syndrome. Reversal by macrophage depletion. *American Journal of Respiratory and Critical Care Medicine*.

[12] Tian W., Jiang X., Tamosiuniene R. (2013). Blocking macrophage leukotriene B_4_ prevents endothelial injury and reverses pulmonary hypertension. *Science Translational Medicine*.

[13] Webster M. K., Goya L., Ge Y., Maiyar A. C., Firestone G. L. (1993). Characterization of sgk, a novel member of the serine/threonine protein kinase gene family which is transcriptionally induced by glucocorticoids and serum. *Molecular and Cellular Biology*.

[14] Lang F., Bohmer C., Palmada M., Seebohm G., Strutz-Seebohm N., Vallon V. (2006). (Patho)physiological significance of the serum- and glucocorticoid-inducible kinase isoforms. *Physiological Reviews*.

[15] Lang F., Stournaras C. (2013). Serum and glucocorticoid inducible kinase, metabolic syndrome, inflammation, and tumor growth. *Hormones*.

[16] Meng F., Yamagiwa Y., Taffetani S., Han J., Patel T. (2005). IL-6 activates serum and glucocorticoid kinase via p38alpha mitogen-activated protein kinase pathway. *American Journal of Physiology. Cell Physiology*.

[17] BelAiba R. S., Djordjevic T., Bonello S. (2006). The serum- and glucocorticoid-inducible kinase Sgk-1 is involved in pulmonary vascular remodeling: role in redox-sensitive regulation of tissue factor by thrombin. *Circulation Research*.

[18] Fagerli U. M., Ullrich K., Stühmer T. (2011). Serum/glucocorticoid-regulated kinase 1 (*SGK1*) is a prominent target gene of the transcriptional response to cytokines in multiple myeloma and supports the growth of myeloma cells. *Oncogene*.

[19] Rotte A., Pasham V., Eichenmuller M., Yang W., Bhandaru M., Lang F. (2011). Influence of dexamethasone on na^+^/h^+^ exchanger activity in dendritic cells. *Cellular Physiology and Biochemistry : International Journal of Experimental Cellular Physiology, Biochemistry, and Pharmacology*.

[20] Wu C., Yosef N., Thalhamer T. (2013). Induction of pathogenic T_H_17 cells by inducible salt- sensing kinase SGK1. *Nature*.

[21] Burgon J., Robertson A. L., Sadiku P. (2014). Serum and glucocorticoid-regulated kinase 1 regulates neutrophil clearance during inflammation resolution. *Journal of Immunology*.

[22] Baban B., Liu J. Y., Mozaffari M. S. (2014). SGK-1 regulates inflammation and cell death in the ischemic-reperfused heart: pressure-related effects. *American Journal of Hypertension*.

[23] Borst O., Schaub M., Walker B. (2015). Pivotal role of serum- and glucocorticoid-inducible kinase 1 in vascular inflammation and atherogenesis. *Arteriosclerosis, Thrombosis, and Vascular Biology*.

[24] Yang M., Zheng J., Miao Y. (2012). Serum-glucocorticoid regulated kinase 1 regulates alternatively activated macrophage polarization contributing to angiotensin II-induced inflammation and cardiac fibrosis. *Arteriosclerosis, Thrombosis, and Vascular Biology*.

[25] Cheng J., Wang Y., Ma Y. (2010). The mechanical stress-activated serum-glucocorticoid-regulated kinase 1 contributes to neointima formation in vein grafts. *Circulation Research*.

[26] Wang J., Fu X., Yang K. (2015). Hypoxia inducible factor-1-dependent up-regulation of BMP4 mediates hypoxia-induced increase of TRPC expression in PASMCs. *Cardiovascular Research*.

[27] Lake F. R., Noble P. W., Henson P. M., Riches D. W. (1994). Functional switching of macrophage responses to tumor necrosis factor-alpha (TNF alpha) by interferons. Implications for the pleiotropic activities of TNF alpha. *The Journal of Clinical Investigation*.

[28] Price L. C., Wort S. J., Perros F. (2012). Inflammation in pulmonary arterial hypertension. *Chest*.

[29] Savai R., Pullamsetti S. S., Kolbe J. (2012). Immune and inflammatory cell involvement in the pathology of idiopathic pulmonary arterial hypertension. *American Journal of Respiratory and Critical Care Medicine*.

[30] Ito T., Okada T., Miyashita H. (2007). Interleukin-10 expression mediated by an adeno-associated virus vector prevents monocrotaline-induced pulmonary arterial hypertension in rats. *Circulation Research*.

[31] Chen G., Zuo S., Tang J. (2018). Inhibition of CRTH2-mediated Th2 activation attenuates pulmonary hypertension in mice. *The Journal of Experimental Medicine*.

[32] Anwar A., Ruffenach G., Mahajan A., Eghbali M., Umar S. (2016). Novel biomarkers for pulmonary arterial hypertension. *Respiratory Research*.

[33] Soon E., Holmes A. M., Treacy C. M. (2010). Elevated levels of inflammatory cytokines predict survival in idiopathic and familial pulmonary arterial hypertension. *Circulation*.

[34] Voelkel N. F., Tamosiuniene R., Nicolls M. R. (2016). Challenges and opportunities in treating inflammation associated with pulmonary hypertension. *Expert Review of Cardiovascular Therapy*.

[35] Lang F., Artunc F., Vallon V. (2009). The physiological impact of the serum and glucocorticoid-inducible kinase SGK1. *Current Opinion in Nephrology and Hypertension*.

[36] Gan W., Li T., Ren J., Li C., Liu Z., Yang M. (2018). Serum-glucocorticoid-regulated kinase 1 contributes to mechanical stretch-induced inflammatory responses in cardiac fibroblasts. *Molecular and Cellular Biochemistry*.

[37] Lang F., Perrotti N., Stournaras C. (2010). Colorectal carcinoma cells--regulation of survival and growth by SGK1. *The International Journal of Biochemistry & Cell Biology*.

[38] David S., Kalb R. G. (2005). Serum/glucocorticoid‐inducible kinase can phosphorylate the cyclic AMP response element binding protein, CREB. *FEBS Letters*.

[39] Zhang L., Cui R., Cheng X., Du J. (2005). Antiapoptotic effect of serum and glucocorticoid-inducible protein kinase is mediated by novel mechanism activating I{kappa}B kinase. *Cancer Research*.

[40] Murakami Y., Hosoi F., Izumi H. (2010). Identification of sites subjected to serine/threonine phosphorylation by SGK1 affecting N-myc downstream-regulated gene 1 (NDRG1)/Cap43-dependent suppression of angiogenic CXC chemokine expression in human pancreatic cancer cells. *Biochemical and Biophysical Research Communications*.

[41] He C., Carter A. B. (2015). The metabolic prospective and redox regulation of macrophage polarization. *Journal of Clinical & Cellular Immunology*.

[42] Cracowski J. L., Chabot F., Labarere J. (2014). Proinflammatory cytokine levels are linked to death in pulmonary arterial hypertension. *The European Respiratory Journal*.

[43] Steiner M. K., Syrkina O. L., Kolliputi N., Mark E. J., Hales C. A., Waxman A. B. (2009). Interleukin-6 overexpression induces pulmonary hypertension. *Circulation Research*.

[44] Fujita M., Mason R. J., Cool C., Shannon J. M., Hara N., Fagan K. A. (2002). Pulmonary hypertension in TNF-*α*-overexpressing mice is associated with decreased VEGF gene expression. *Journal of Applied Physiology*.

[45] Savale L., Tu L., Rideau D. (2009). Impact of interleukin-6 on hypoxia-induced pulmonary hypertension and lung inflammation in mice. *Respiratory Research*.

[46] Voelkel N. F., Tuder R. M., Bridges J., Arend W. P. (1994). Interleukin-1 receptor antagonist treatment reduces pulmonary hypertension generated in rats by monocrotaline. *American Journal of Respiratory Cell and Molecular Biology*.

